# Extensive structural variation in the Bowman-Birk inhibitor family in common wheat (*Triticum aestivum* L.)

**DOI:** 10.1186/s12864-021-07475-8

**Published:** 2021-03-25

**Authors:** Yucong Xie, Karl Ravet, Stephen Pearce

**Affiliations:** Department of Soil and Crop Sciences, Colorado State University, Fort Collins, CO 80523 USA

**Keywords:** Protease inhibitor, Bowman-Birk inhibitor, Tandem duplication, Biotic stress, Wheat

## Abstract

**Background:**

Bowman-Birk inhibitors (BBI) are a family of serine-type protease inhibitors that modulate endogenous plant proteolytic activities during different phases of development. They also inhibit exogenous proteases as a component of plant defense mechanisms, and their overexpression can confer resistance to phytophagous herbivores and multiple fungal and bacterial pathogens. Dicot BBIs are multifunctional, with a “double-headed” structure containing two separate inhibitory loops that can bind and inhibit trypsin and chymotrypsin proteases simultaneously. By contrast, monocot BBIs have a non-functional chymotrypsin inhibitory loop, although they have undergone internal duplication events giving rise to proteins with multiple BBI domains.

**Results:**

We used a Hidden Markov Model (HMM) profile-based search to identify 57 BBI genes in the common wheat (*Triticum aestivum* L.) genome. The BBI genes are unevenly distributed, with large gene clusters in the telomeric regions of homoeologous group 1 and 3 chromosomes that likely arose through a series of tandem gene duplication events. The genomes of wheat progenitors also contain contiguous clusters of BBI genes, suggesting this family underwent expansion before the domestication of common wheat. However, the BBI gene family varied in size among different cultivars, showing this family remains dynamic. Because of these expansions, the BBI gene family is larger in wheat than other monocots such as maize, rice and *Brachypodium*.

We found BBI proteins in common wheat with intragenic homologous duplications of cysteine-rich functional domains, including one protein with four functional BBI domains. This diversification may expand the spectrum of target substrates. Expression profiling suggests that some wheat BBI proteins may be involved in regulating endogenous proteases during grain development, while others were induced in response to biotic and abiotic stresses, suggesting a role in plant defense.

**Conclusions:**

Genome-wide characterization reveals that the BBI gene family in wheat is subject to a high rate of homologous tandem duplication and deletion events, giving rise to a diverse set of encoded proteins. This information will facilitate the functional characterization of individual wheat BBI genes to determine their role in wheat development and stress responses, and their potential application in breeding.

**Supplementary Information:**

The online version contains supplementary material available at 10.1186/s12864-021-07475-8.

## Background

Plant proteases play vital roles in diverse biological processes by modulating programmed cell death, nutrient remobilization and defense responses [1]. Their activity is regulated by different classes of protease inhibitors (PIs) which bind to their protease substrates either through an irreversible trapping reaction or a tight-binding reaction [2–4]. In plants, PIs regulate the activity of endogenous proteases to prevent proteolytic degradation, for example, by controlling the mobilization of storage proteins in seeds and kernels, and regulating senescence [5, 6]. They also play important roles in plant defense by regulating the activity of exogenous proteases from different types of pests and pathogens to prevent cellular damage [7]. In response to insect feeding, plant PIs are released into the insect’s guts and inhibit digestive protease enzymes, which can prevent nutrient absorption, retarding their growth and development [8]. Plant PIs are also induced by effector triggered immunity in response to bacterial and fungal pathogens to inhibit their proteolytic enzymes [9–11]. PIs are categorized into four broad classes according to their target protease specificity: serine PI (serpins), cysteine PI (cystatins), aspartic acid PI (pepstatins), and metallo-carboxy PI [2]. PIs are further classified into types, families and clans to reflect their evolutionary relationships based on sequence homology, structural variation and biochemical function [12–14]. The latest PI classifications are maintained in the MEROPS database [15].

Bowman-Birk inhibitors (BBIs) are a family of serine-type PIs in MEROPS family I12, clan IF, that inhibit trypsin and chymotrypsin protease activity via the tight-binding reaction mechanism [16, 17]. Members of the BBI family are best known for their role in plant defense against phytophagous insects, and have been used to engineer insect-resistant transgenic crops [18]. Overexpression of a cowpea trypsin inhibitor gene, which encodes a BBI protein, confers resistance to insects in the orders Coleoptera and Lepidoptera in tobacco [19], rice [20], and wheat [21]. Several BBI proteins also exhibit trypsin-like protease inhibition against fungal pathogens including *Mycosphaerella arachidicola, Fusarium oxysporum*, and *Botrytis cinerea* [22, 23], *Fusarium culmorum* [24] and *Pyricularia oryzae* [25]*,* as well as bacterial pathogens such as *Xanthomonas oryzae* pv*. Oryzae* [26]. One rice BBI, APIP4, interacts at the protein level with both a fungal effector and host NLR receptors as part of the innate immune response, and plants carrying loss-of-function mutations in this gene exhibit increased susceptibility to *Magnaporthe oryzae* [27]. In wheat, genetic mapping studies identified putative BBI genes as candidates for seedling resistance to tan spot [28] and Fusarium head blight [29]. There is also evidence that BBIs play roles in more diverse processes, such as tolerance to salinity [30], oxidative [31], and drought stress [32, 33], and regulating Fe uptake via an unknown mechanism [34].

First discovered in soybean in 1946 [35], BBIs had until recently only been described in the Fabaceae and Poaceae families [36]. The BBIs are now known to be widely distributed in angiosperms [36–38], and evolutionary and phylogenetic analyses suggest they share a common ancestral sequence [38]. The characterization of five BBIs in *Selaginella moellendorffii,* the oldest known extant vascular plant, show that this ancestral protein has a characteristic “double-headed” structure with two homologous and spatially separated inhibitory loops within one BBI domain [38]. Conserved inhibitory loops form reactive motifs providing dual specificity [36]. BBI domains are also characterized by a series of conserved Cysteine (Cys) residues, which form disulfide bridges to provide structural stability required to maintain inhibitory loop conformation [36, 37]. The mutation of a single conserved Cys residue forming a disulfide bridge is sufficient to abolish the activity of either inhibitory loop [39], and BBI domains with fewer than ten Cys residues are predicted to be non-functional [36]. The Cys-formed inhibitory loops contain reactive domains composed of variable amino acids responsible for binding to trypsin and to chymotrypsin, including two residues, P1 and P1’, that are proposed to play a role in determining protease substrate specificity [36]. BBI proteins also commonly have a hydrophobic signal peptide (SP) at their N-terminus, with high sequence diversity among different BBIs [40, 41]. The SP is required for BBI protein translocation and secretion into the extracellular space, although it is not necessary for protease inhibition since the inhibitory loops can function independently of the rest of the BBI protein [42]. There is also evidence that BBI proteins can act in the nucleus [34]. All characterized BBI proteins in dicotyledonous plants have a conserved “double-headed” structure with a consistent molecular weight of approximately 8 kDa [36–38, 43].

By contrast, almost all BBIs in monocotyledonous plants lack conserved Cys residues in the second inhibitory loop that are required to inhibit chymotrypsin, leading to a “single-headed” structure so that each BBI domain consists of only one functional reactive loop to inhibit trypsin activity [36]. The only known exceptions are three “double-headed” BBIs in the banana (*Musa acuminate*) genome, indicating that the “single-headed” BBI structure originated since the monocot and dicot lineages diverged [38]. Evolutionary models indicate that monocot BBIs underwent internal domain duplications within a single protein that resulted in multiple inhibitory loops [25, 36, 44]. Previous studies divided monocot BBI proteins into six groups (﻿MI-I to MI-VI) on the basis of their functional domain number and the number and position of conserved Cys residues [10, 36, 45]. To simplify, these six BBI models in monocots can be grouped into three broad classes; one comprised of 8 kDa proteins with a single functional domain (groups MI-I, MI-II, and MI-III), a second class with a molecular weight of approximately 16 kDa and a duplicated single-inhibitory loop (groups MI-IV and MI-V), and a final category of larger proteins with three tandemly duplicated BBI domains. While the first two classes are widespread in monocots, only three rice BBIs have been described which fall into the final class [25].

Genome-wide studies of the BBI gene family have been performed in rice [25], common bean [46] and other angiosperms [38]. However, to date, only three BBIs have been characterized in common wheat (*Triticum aestivum* L.), a crop which provides approximately 20% of the calories and proteins consumed by the human population [47]. Of the three BBI proteins isolated from wheat germ, IBB1 has two homologous functional domains, each with one functional inhibitory loop [48, 49], whereas IBB2 and IBB3 have only one functional domain [48, 50]. These three BBIs inhibit protease activity, control protein metabolism during wheat kernel development and germination, and inhibit fungal trypsin-like activity and hyphal growth [51]. Three other putative genes with sequence homology to BBIs (*wali3, wali5,* and *wali6*) were isolated as cDNAs from wheat root tips [30, 52, 53]. These putative BBI genes are transcriptionally induced by wounding or by the imposition of toxic metal stress, but their function against protease was not tested [30, 53].

The identification of wheat BBI genes is complicated by the high frequency of residue substitution and sequence variability among encoded proteins, and the complexity of the wheat genome. Common wheat is an allopolyploid (genomes AABBDD) produced from two separate hybridization events. The first occurred approximately 0.5 to 0.9 million years ago between *T. urartu* (AA) and an unknown species related to *Aegilops speltoides* to form the tetraploid wild emmer wheat *T. turgidum* ssp. *dicoccoides* (AABB). A second hybridization event between *T. turgidum* ssp. durum and *Ae. tauschii* (DD) gave rise to common wheat, approximately 10,000 years ago [54].

In the current study, we used a Hidden Markov Model (HMM)-based approach to describe the BBI gene family in common wheat, revealing it to be larger than in other monocot species. We found evidence of extensive gene duplications throughout wheat’s evolutionary history, as well as internal duplications that further diversified the functional BBI domains of individual proteins. The findings from our study highlight the extent of variation in the BBI gene family in the Triticeae lineage and will facilitate their functional characterization to explore how this diversity impacts wheat development and plant defense.

## Results

### Bowman-Birk inhibitor genes are unevenly distributed in the common wheat genome

**Fig. 1:**
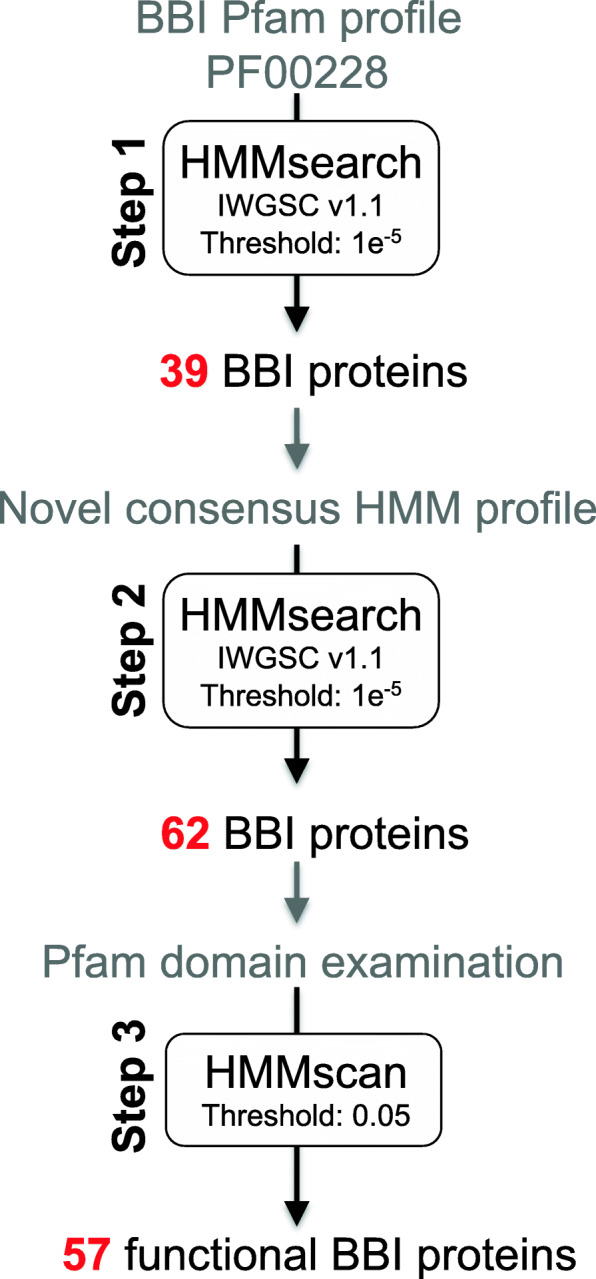
Pipeline for Bowman-Birk inhibitor (BBI) gene family identification in plant genomes. The identification of BBIs in the *T. aestivum* genome is presented as an example, including key steps and criteria for each step. The number of proteins identified at each stage are highlighted in red

We identified 57 BBI genes in the hexaploid common wheat genome using a three-step HMM-based approach outlined in Fig. 1. We first used the HMM profile for BBI (Pfam: PF00228, downloaded from the Pfam database) to search the IWGSC RefSeq v1.1 protein database and identified 39 BBI proteins. We generated a new HMM profile based on the alignment of these 39 sequences and used this in a second search against the same protein database to identify 62 BBI proteins, including 23 that were not found in the first step. We performed HMMscan on each protein and excluded five sequences that lacked a BBI Pfam domain (Additional file 2, Table S1). A final search using an HMM profile built from an alignment of the remaining 57 BBIs did not yield any additional proteins, confirming this is a comprehensive list of annotated BBI proteins in the wheat landrace ‘Chinese Spring’ (Additional file 2, Table S1).

We manually adjusted the start codon position for five BBIs to match homologous sequences (Additional file 2, Table S2). After manual curation, 50 full-length BBIs are predicted to have an N-terminal SP domain, with cleavage positions ranging from 15 to 30 amino acids. Seven N-terminally truncated BBIs are predicted to lack a functional SP domain (Additional file 2, Table S1).

The 57 BBIs include three genes (*TraesCS3A02G046000*, *TraesCS3B02G036400*, and *TraesCS1B02G025900*) that encode previously characterized BBI proteins - IBB1, IBB2, and IBB3 (Additional file 2, Table S3) [48, 50]. Three other previously described putative BBI genes (*wali3, wali5* and *wali6* [52, 53]) were not found among the 57 BBIs. An HMMscan analysis of the corresponding full-length proteins (TraesCS1D02G265900, TraesCS1D02G265800 and TraesCS1B02G276900) revealed that they did not contain a BBI domain, indicating these genes do not encode functional BBI proteins (Additional file 2, Table S3).

**Fig. 2:**
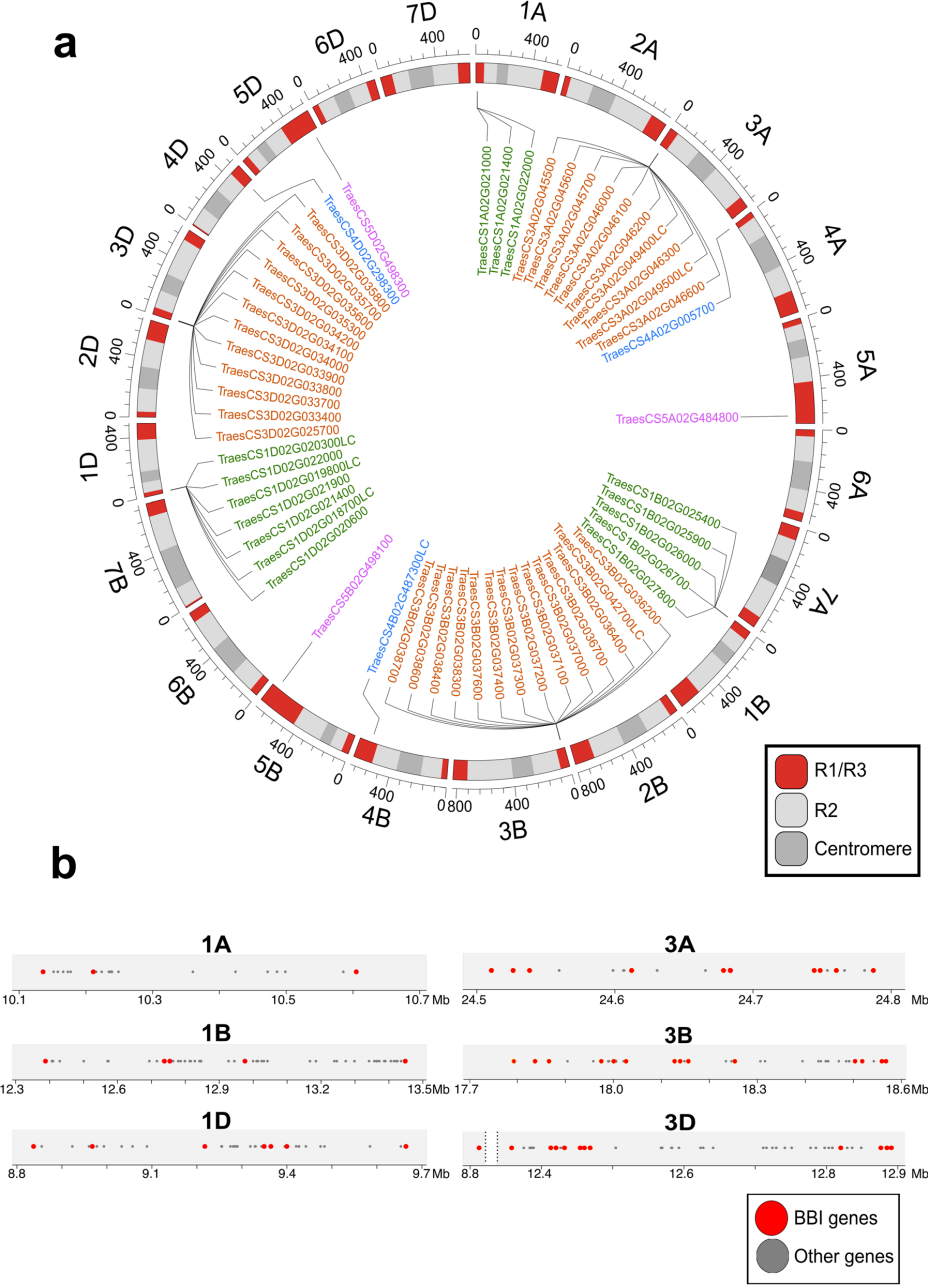
Distribution of 57 BBI in the *T. aestivum* genome. **a** Chromosomal positions of wheat BBIs. Gene names are colored according to their homoeologous group. Chromosomal segments are indicated by different colors - distal regions of the chromosome R1 and R3 in red, centromeric region C in dark grey, and region R2 in light grey. **b** Distribution of genes within BBI clusters on homoeologous group 1 and group 3 chromosomes. Red dots represent BBI genes, whereas grey dot represent other annotated genes in the region, positioned according to their physical location in the IWGSC Refseq v1.1 genome assembly. All high confidence (HC) and low confidence (LC) gene models are presented

Wheat BBI genes are unevenly distributed across the genome with two gene triads on chromosomes 4 and 5 and large clusters on homoeologous group 3 (36 BBIs) and group 1 chromosomes (15 BBIs) (Fig. 2a, b). The BBI genes in these clusters are separated by short physical distances and in several instances include adjacent BBIs, suggesting they arose through tandem gene duplication events (Fig. 2a, b). For example, the ten BBIs on chromosome 3A span a region of just 270 kb and include four adjacent BBIs (Fig. 2b). All wheat BBIs were located in the telomeric regions (R1 and R3) of their respective chromosomes (Fig. 2a).

This pattern of gene duplication is consistent with homology analysis that divided the 57 BBIs into six homoeologous categories (Table 1). Overall, 21 BBI genes (36.8% of the total) formed seven complete triads (1:1:1 for A:B:D genome), close to the 35.8% for all wheat genes in the genome [55]. By contrast, 14% of BBI genes form groups characterized by gene duplication (n:1:1/1:n:1/1:1:n) compared to 5.7% of all wheat genes [55] (Table 1). In addition, one group of genes consisted of four tandemly duplicated genes on chromosome 1B (0:4:0), while on chromosome 3, one group exhibited duplications of both the A and B homoeologs (2:2:1) (Table 1; Additional file 2, Table S4).

**Table 1:** Homoeologous group identification and categorization of the BBI gene family in wheat

Category number	Homoeologous group (A:B:D)	Number of groups	Number of genes	% of genes
1	1:1:1	7	21	36.8
2	2:1:1 and 1:2:1	2	8	14
3	1:1:0 and 0:1:1	2	4	7
4	0:4:0	1	4	7
5	2:2:1 and 2:0:2	2	9	15.8
6	Singletons	11	11	19.4
–	Total	25	57	100

To determine whether these duplication events affected the selective pressure on BBI genes, we performed a Ka/Ks ratio analysis to calculate the sequence divergence rate for the clusters of BBIs on individual homoeologous group 1 and 3 chromosomes. A ratio of non-synonymous (Ka) to synonymous (Ks) nucleotide changes greater than one indicates divergent function of two genes, whereas a Ka/Ks ratio of less than one indicates purifying selection and conserved function. The Ka/Ks ratios for pairwise comparisons of BBI genes on homoeologous group 1 chromosomes were all less than one, except for one branch on chromosome 1D between *TraesCS1D02G020600* and *TraesCS1D02G018700LC* that had a value of 1.17 (Additional file 1, Fig. S1). By contrast, eight branches on homoeologous group 3 chromosomes had Ka/Ks values greater than one, including four branches on 3A, two branches on 3B, and two branches on 3D (Additional file 1, Fig. S1).

Overall, our analysis shows that the BBI family in wheat is unevenly distributed across the genome and includes large gene clusters in the telomeric regions of homoeologous group 1 and group 3 chromosomes. The distribution of the genes in these clusters suggest they originated from paralogous expansion through tandem duplication events.

### BBI genes underwent extensive tandem duplications in the Triticeae

**Fig. 3:**
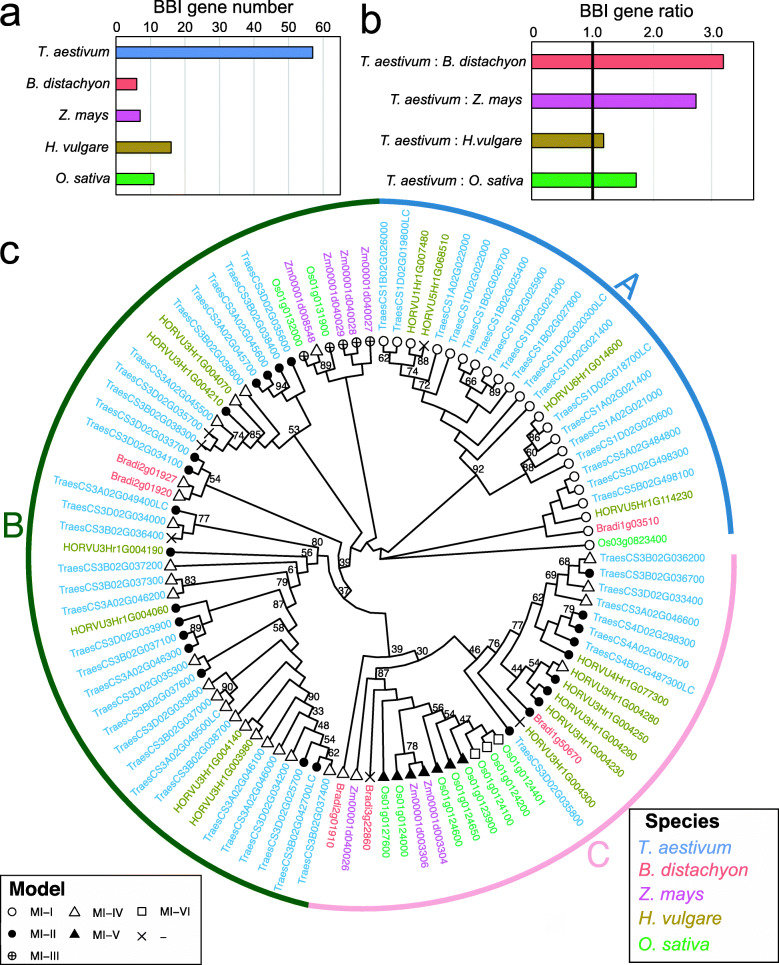
Comparison of the wheat BBI gene family with other monocots. **a** Total number of BBI genes in monocot genomes. Bars are color-coded based on species. **b** Ratios of total BBI gene numbers in common wheat compared to other monocot species, adjusted for wheat’s hexaploid genome. The 1:1 ratio is indicated by a bold line. **c** Circular phylogenetic tree of all BBI proteins from rice, maize, barley, *Brachypodium* and common wheat. Only bootstrap support values below 95 are indicated on the tree. Gene labels are color-coded by species and includes the BBI group based on the classification of Mello et al. [36]

We next compared the BBI family in wheat with other monocot species. Using the same approach and criteria (Fig. 1), we identified six BBIs from *Brachypodium* (*B. distachyon*), seven from maize (*Z. mays*), eleven from rice (*O. sativa*), and sixteen from barley (*H. vulgare*) (Fig. 3a). A full list of BBIs from each species is provided in Additional file 2, Table S5. Considering its hexaploid genome, common wheat has an average of 19 BBI genes per diploid genome, 3.2-fold more than *Brachypodium*, 2.7-fold more than maize, 1.7-fold more than rice, but just 1.2-fold more than barley (Fig. 3b).

To explore the genetic relationships between BBIs in these species, we constructed a phylogenetic tree from all identified proteins. The tree separated wheat BBIs into three broad clades, each of which also contained BBIs from other species, except clade A that does not contain maize BBIs (Fig. 3c). Clade A clustered all wheat BBIs located on homoeologous group 1 and 5 chromosomes. Clade B included the majority of wheat BBIs located on homoeologous group 3 chromosomes, with the remainder clustered in clade C together with the BBI gene triad from chromosome 4 (Fig. 3c).

Consistent with their relatively recent divergence and the similarity in size of the BBI gene family, most barley BBIs co-located with wheat BBIs (Fig. 3c). However, one cluster of contiguous BBIs on barley chromosome 3H suggests that gene duplication events also occurred independently in this species (Clade C, Fig. 3c). Maize and rice BBIs formed two distinct clusters in clade B and clade C, which included several adjacent BBIs in their respective genome assemblies, suggesting that BBI gene duplication also occurred independently in both these species (Fig. 3c).

BBI proteins were also separated according to the type of reactive site and the number of active domains they contained, as defined by Mello et al. [36]. Every BBI from all species in clade A contains a single active BBI domain and all fall into the MI-I group except for one barley BBI (HORVU5Hr1G068510) that does not match any previously characterized BBI group (Fig. 3c). The wheat BBIs on chromosome 3 clustered in clades B and C are all multi-domain proteins, and fall into either the MI-II or MI-IV groups except for three wheat BBIs with more than two domains that are most similar to the MI-IV group (Fig. 3c). The cluster of rice, maize and *Brachypodium* BBIs in clade C were most similar to the wheat BBIs on homoeologous group 3 chromosomes, and were also all multi-domain proteins, represented by groups MI-IV, MI-V and MI-VI (Fig. 3c).

This phylogeny reveals that the BBI gene family in monocots is subject to a complex pattern of internal and external gene duplication events, resulting in multi-domain BBIs and gene copy number variation in each species. In wheat, extensive gene duplication on homoeologous group 1 and especially group 3 chromosomes, that also occurred in barley, account for the greater numbers of BBI genes in the Triticeae lineage compared to other grasses.

### The BBI gene family underwent gene duplication and deletion events both before and after common wheat’s domestication

**Fig. 4:**
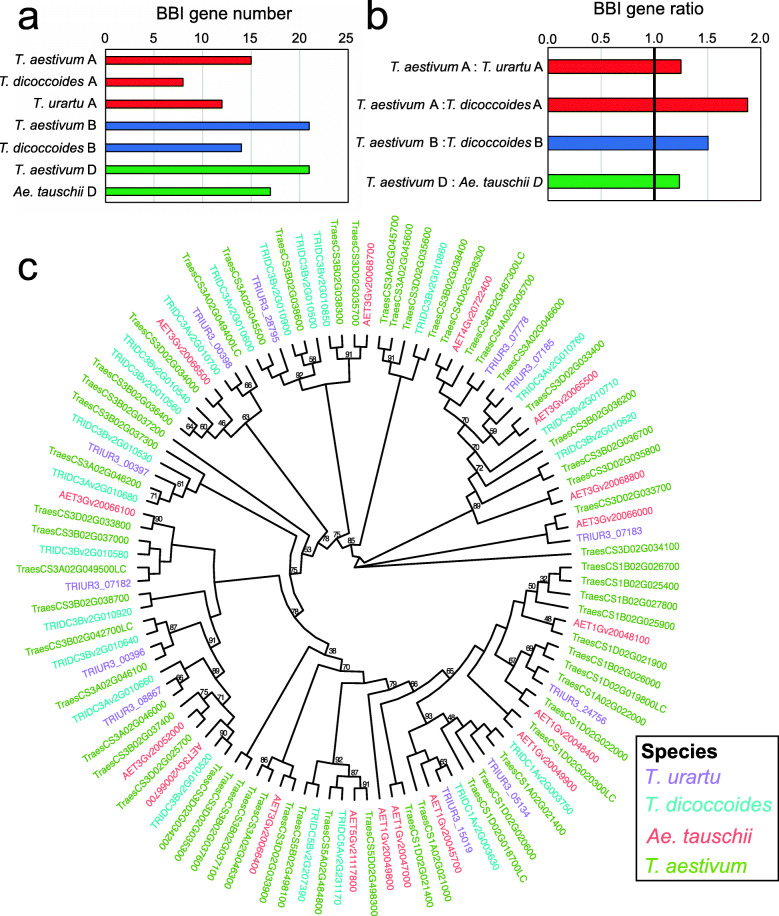
Comparison of the BBI gene family in different wheat germplasm. **a** The number of BBI genes in the genomes of different wheat species. Bars are color coded by species. **b** Ratio of total BBI gene numbers in common wheat compared to progenitor species. The 1:1 ratio is indicated by a bold line. **c** Phylogenetic tree constructed from all BBI proteins from each wheat species. Only bootstrap support values below 95 are indicated on the tree. Genes are color-coded based on species

To gauge the approximate timing of the BBI gene family expansion in wheat, we identified BBI proteins from common wheat’s ancestors. We found 12 BBIs from *T. urartu* and 17 from *Ae. tauschii*, the diploid progenitors of the A and D genomes of common wheat, respectively (Fig. 4a). Because the diploid wheat B genome progenitor is unknown, we analyzed *T. dicoccoides*, an allotetraploid progenitor with genomes AABB, and identified 23 BBIs. We excluded one of these genes from our analysis (*TRIDCUv2G007850*) because it was not assembled into a known chromosome, leaving eight BBIs on the A genome and fourteen on the B genome (Fig. 4a). Compared to each diploid progenitor genome, the corresponding genome in *T. aestivum* contained a greater number of BBIs (Fig. 4b). There were 1.3-fold more BBIs on the A genome of *T. aestivum* than in *T. urartu* and 1.9-fold more genes than in the A genome of *T. dicoccoides* (Fig. 4b). There were 1.5-fold more BBIs on the B genome of *T. aestivum* compared to *T. dicoccoides*. By contrast, the *T. aestivum* D genome contains only 1.2-fold more BBI genes than *Ae. tauschii* (Fig. 4b).

Phylogeny showed that most genes from wheat ancestors were clustered into orthologous groups with their corresponding genes in common wheat (Fig. 4c, Additional file 2, Table S6). Orthologs of the BBI genes on *T. aestivum* homoeologous group 4 chromosomes were present in *T. urartu* (AA genome) and *Ae. tauschii* (DD genome), but absent from *T. dicoccoides* (AABB genomes). Orthologs of the BBI genes on *T. aestivum* group 5 chromosomes were present in both A and B genomes of *T. dicoccoides* and in the D genome of *Ae. tauschii* (Additional file 2, Table S6). None of these genes were duplicated in any wheat species.

By contrast, the similarity and genomic position of BBI gene clusters on homoeologous group 1 and 3 chromosomes in progenitor wheat species suggests that many BBI gene duplication events occurred before common wheat’s domestication. On *Ae. tauschii* chromosome 1D, six contiguous BBI genes are clustered within 800 kb, while on chromosome 3D, eight BBI genes are clustered within 500 kb, suggesting they arose through tandem duplication (Additional file 2, Table S5). In *T. dicoccoides*, there are five BBI genes on chromosome 3A within a 264 kb region and thirteen BBI genes on chromosome 3B within a 696 kb region (Additional file 2, Table S5).

This phylogeny also revealed several instances of gene duplications in hexaploid *T. aestivum* that were absent in the diploid or tetraploid progenitors. For example, we found a cluster of four adjacent paralogous BBIs on chromosome 1B of *T. aestivum* that were all absent from *T. dicoccoides,* suggesting that tandem duplication events occurred after common wheat’s domestication (Fig. 4c and Additional file 2, Table S6).

**Fig. 5:**
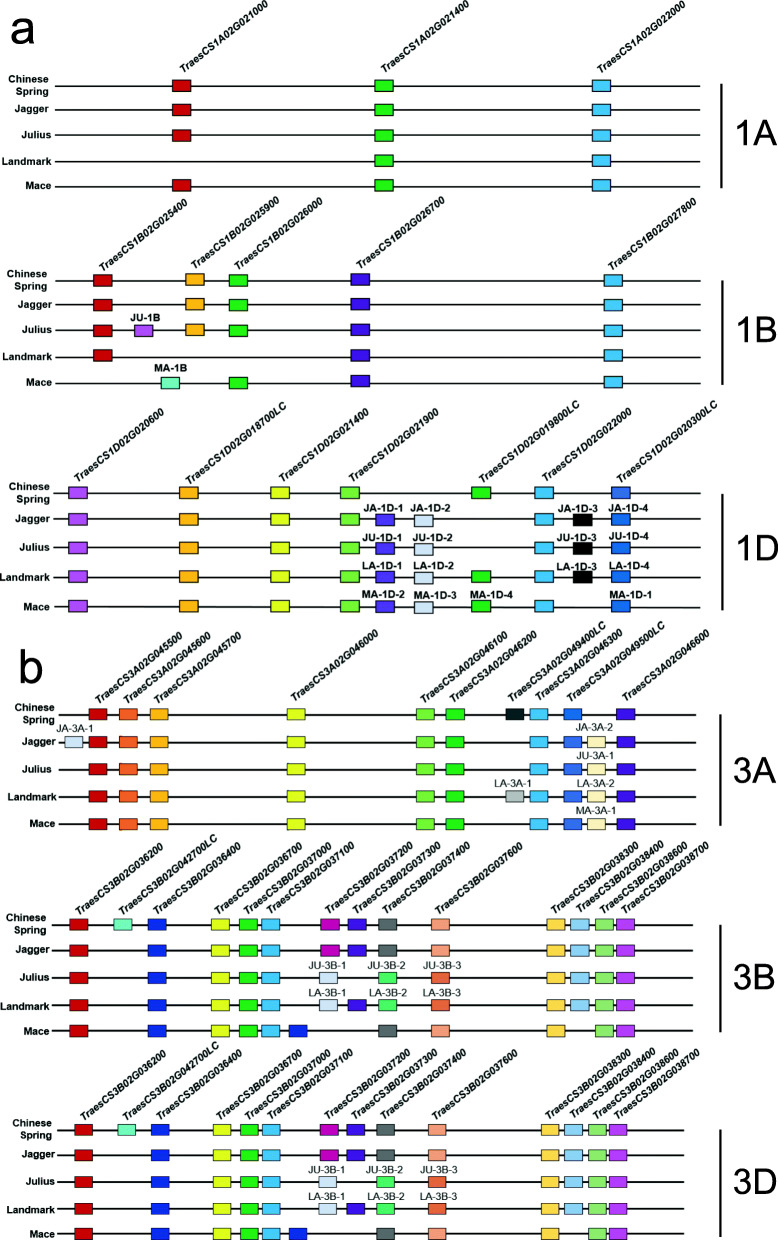
Distribution of BBI genes on homoeologous group 1 and 3 chromosomes in different common wheat varieties. **a**. Homoeologous group 1 chromosomes. **b**. Homoeologous group 3 chromosomes. The ‘Chinese Spring’ BBIs are ordered according to their physical position in the IWGSC RefSeq v.1.1 genome assembly, but not to scale. Genes are colored according to their homology so that genes in the same color are orthologous in different varieties. The BBIs present in other varieties but absent in ‘Chinese Spring’ are labeled such that JA_3A-1 indicates the first unique BBI on ‘Jagger’ chromosome 3A

To analyze the diversity within the BBI gene family arising from selections made during domestication and breeding, we identified BBIs in the genome assemblies of four common wheat cultivars (Additional file 1, Table S7). The total number of BBI genes in these cultivars ranged from 55 in ‘Mace’ to 60 in ‘Jagger’ (Table 2). While the BBI gene triads on chromosomes 4 and 5 were conserved in all cultivars, phylogenetic analysis indicated several instances of gene loss and gain on homoeologous group 1 and 3 chromosomes (Additional file 1, Fig. S2). Although the BBI gene number varied between cultivars on each of these chromosomes, this variation was greatest on chromosomes 1B, 1D and 3B (Fig. 5a, b, Table 2). Strikingly, none of the five analyzed cultivars shared an identical complement of BBI genes.

**Table 2:** BBI genes in five common wheat varieties, separated by chromosome

Chromosome	Chinese Spring	Jagger	Mace	Julius	Landmark	***∆***_***max − min***_
1A	3	3	3	3	2	1
1B	5	5	4	6	3	3
1D	7	9	9	9	10	3
3A	10	11	10	10	11	1
3B	14	13	11	12	13	3
3D	12	13	12	13	13	1
4A	1	1	1	1	1	0
4B	1	1	1	1	1	0
4D	1	1	1	1	1	0
5A	1	1	1	1	1	0
5B	1	1	1	1	1	0
5D	1	1	1	1	1	0
Total	57	60	55	59	58	5

Δ_*max* − *min*shows the inter-varietal variation in BBI gene number for each chromosome_

Taken together, analysis of the BBI gene family in different wheat germplasm reveals that while the gene triads on chromosomes 4 and 5 did not undergo expansion throughout wheat evolution, the gene clusters on homoeologous group 1 and 3 chromosomes are more variable. Many gene duplication events occurred before domestication, but the increase in gene number in common wheat and variation among modern wheat cultivars shows that the BBI family remains dynamic.

### Wheat BBI genes on homoeologous group 3 chromosomes encode proteins with duplicated active domains

**Fig. 6:**
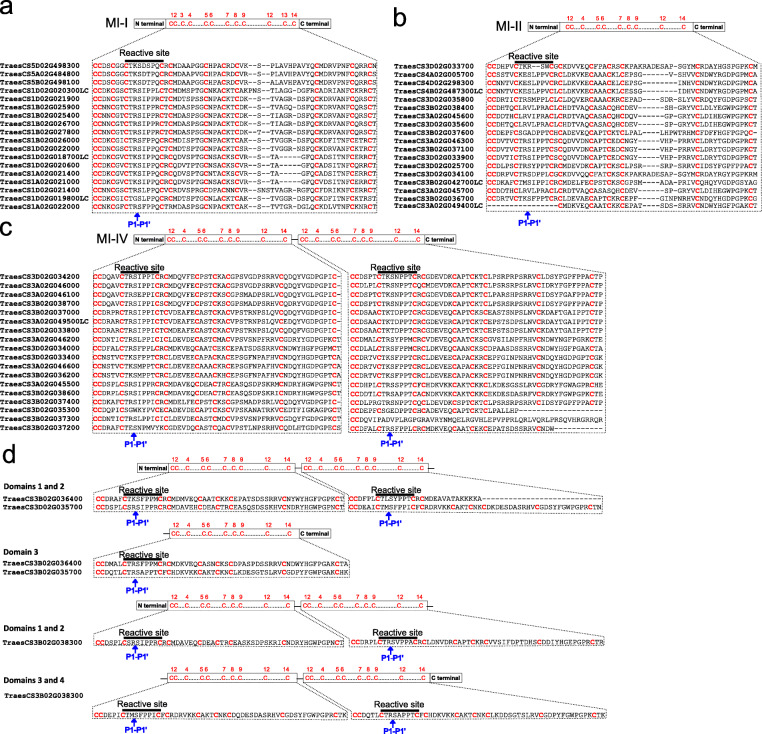
Alignment of the conserved Cys-rich domains of common wheat BBI proteins. **a** Alignment of common wheat BBI proteins falling into group MI-I; **b** MI-II group; **c** MI-IV group; and **d** BBI proteins that cannot be classified into an existing group. The Cys residues are highlighted in red with their corresponding position indicated above each alignment. The blue arrow underneath the domain sequences highlight the P1 and P1’ positions

We next studied in greater detail the functional domains in the 57 BBIs from ‘Chinese Spring’. The majority of wheat BBIs (36 proteins, 63%) had one functional BBI domain, including all 15 BBIs located on homoeologous group 1 chromosomes, the gene triads on chromosomes 4 and 5 and 15 BBIs on homoeologous group 3 chromosomes (Fig. 6a, b). Of the remaining BBIs on group 3 chromosomes, 18 had two functional BBI domains (Fig. 6c), two proteins (TraesCS3D02G036400 and TraesCS3D02G035700) had three domains and one protein (TraesCS3B02G038300) had four domains (Fig. 6d). The gene structure of wheat BBIs reveals that while the majority have either one (6 BBIs, 10%) or two exons (45 BBIs, 79%), five genes including all three-domain proteins had three exons, while the gene (*TraesCS3B02G038300*) encoding the four-domain protein had four exons (Additional file 1, Fig. S3). This suggests that the genes encoding three- or four-domain BBI proteins may have evolved either from complete or partial gene duplication followed by fusion of tandem-duplicated genes. However, the genomic sequences encoding these domains, including intron and flanking sequences, were variable between domains, suggesting they did not arise from recent duplication events.

We characterized the number and positions of conserved Cys residues within the reactive motifs of wheat BBIs according to the evolutionary scheme of Mello et al. [36] and with respect to substrate specificity. The first reactive inhibitory motif for trypsin was predicted to be conserved and functional in all wheat BBIs except for three proteins: TraesCS3A02G049400LC, which has a truncated motif in a functional BBI domain (Fig. 6b), TraesCS3D02G033700, which has a two-amino acid deletion within the reactive motif (Fig. 6b), and TraesCS3B02G037200, which carries a Cys to Tyrosine (Y) amino acid substitution in the final residue of the first reactive motif (Fig. 6c). The vast majority of wheat BBIs carried K/R-S amino acids at the P1 and P1’ positions, respectively (Fig. 6), the consensus motif for monocot trypsin inhibition [36]. All MI-I type BBIs had a K/R-S motif except one protein (TraesCS1D02G019800LC) that has a Glycine (G) in the P1 residue (Fig. 6a). Among the MI-II type BBIs, the P1-P1’ motif was more diverse. Notably, four homoeologous BBIs on group 3 chromosomes each exhibited Serine (S) to Valine (V) substitutions at position P1’, while each protein in the triad on chromosome 4 had a Glutamate (E) residue at position P1 (Fig. 6b). Most MI-IV type BBIs also had a K/R-S motif in both reactive sites except a homoeologous triad of BBIs with Serine to Tyrosine (Y) substitutions in the P1’ residue in their second domain (Fig. 6c). In all 57 wheat BBIs the disulfide bridge (C_10_ and C_11_) supporting the second inhibitory motif for chymotrypsin was lost (Fig. 6a-d).

**Fig. 7:**
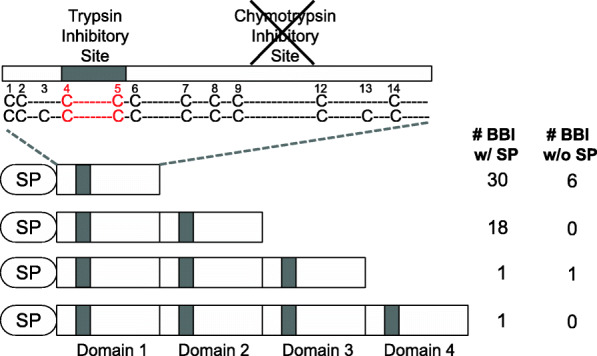
Summary of the proposed structural composition of the BBI gene family in common wheat. Most wheat BBI proteins contain an N-terminal signal peptide, and between one and four reactive loop domains at the C-terminus. The conserved Cys residues in the inhibitory domain are listed as C, and other amino acid residues indicated as dashes. The first reactive site is highlighted in red. The numbers of wheat BBIs from ‘Chinese Spring’ falling into each category are separated on the basis of presence or absence of complete signal peptides

The gene triad on chromosome 5 and all 15 BBIs on homoeologous group 1 chromosomes each encode BBI proteins with functional domains comprised of 12 Cys residues that form six disulfide bridges, except for TraesCS1A02G022000 and TraesCS1A02G019800LC which carry amino acid substitutions at Cys residues in positions C_6_ and C_14_, respectively (Fig. 6a). The homoeologous triad of BBIs on chromosome 4 fall into the MI-II group (Fig. 6b). BBIs on group 3 chromosomes were the most divergent. There were 15 BBIs categorized into the MI-II group that each contain ten Cys residues, except for TraesCS3A02G049400LC which has a deletion encompassing four Cys residues, and TraesCS3A02G045700, TraesCS3B02G042700LC and TraesCS3D02G034100 which each carry a single Cys amino acid substitution (Fig. 6b). Another 18 BBIs encode two-domain proteins categorized in the MI-IV group although three (TraesCS3D02G035300, TraesCS3B02G037300 and TraesCS3B02G03740) had a truncated second domain, while another protein (TraesCS3B02G03720) has fewer than ten Cys residues in both domains (Fig. 6c). The three wheat BBIs with more than two domains could not be categorized into any previously described MI evolutionary group (Fig. 6d). The three-domain proteins TraesCS3B02G036400 and TraesCS3D02G035700 are most similar to the MI-IV group but each underwent internal duplication of one domain resulting in three adjacent BBI domains that have distinct Cys positions from the previously proposed MI-VI three-domain group [36]. TraesCS3B02G036400 has a truncated second domain and a deletion of five Cys residues while the Cys positions in TraesCS3D02G035700 are also divergent from existing models (Fig. 6d). Each of the four domains in TraesCS3B02G038300 are full length and contain ten conserved Cys residues, suggesting all four may be functional (Fig. 6d). In summary, five BBIs (TraesCS3A02G045700, TraesCS3D02G034100, TraesCS3B02G042700LC, TraesCS3A02G049400LC, and TraesCS3B02G03720) have fewer than ten Cys residues in one or both BBI domains, and are predicted to be non-functional. While four other multi-domain BBIs (TraesCS3B02G036400, TraesCS3B02G03730, TraesCS3B02G03740, and TraesCS3D02G035300) have fewer than ten Cys residues in one domain, these proteins are predicted to exhibit protease inhibition activity since at least one other domain remains intact (Fig. 6 and Additional file 2, Table S1). A summary of the different types of wheat BBI proteins in common wheat is shown in Fig. 7.

### Wheat BBI genes exhibit diverse expression profiles during development and in response to biotic and abiotic stress

**Fig. 8:**
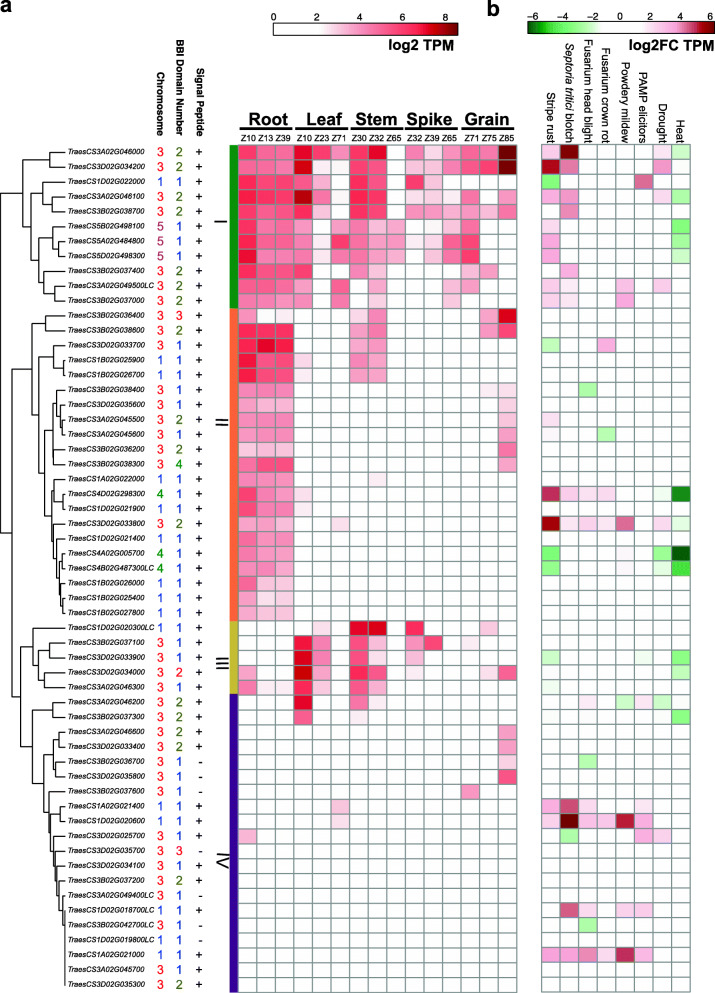
Expression profiles of common wheat BBI genes. **a** Transcript levels of BBI genes in five tissue types (root, leaf, stem, spike and grain) each at three different developmental stages based on the Zadoks scale. Genes were clustered based on their expression profile and all expression data is presented as log2 TPM. **b** Transcript levels of BBI genes in response to biotic and abiotic stress. Expression levels of each BBI gene in wheat plants infected with fusarium head blight, stripe rust, powdery mildew, fusarium crown rot, *Septoria tritici* blotch, and bacterium chitin and flg22 (PAMP) were compared to the corresponding mock treatment. Expression is presented as log2 fold change of the TPM between pathogen treatment and mock control. Drought and heat stress data is taken from one-week-old seedlings and presented as log2 fold change of the TPM between stress and control seedlings. Where multiple timepoints were in each dataset, mean fold-changes are presented. Genes are in the same orders as in the tissue specific developmental conditions in panel a. The number of BBI domains and chromosome for each gene are color-coded. The presence (+) or absence (−) of a signal peptide for each protein is indicated

We next used public RNA-seq datasets to characterize transcript levels of the 57 BBI genes in common wheat [56]. Genes were clustered into four main groups based on their expression profile in different wheat tissues and at different stages of development (Fig. 8a). Genes in group I showed relatively high transcript levels in most plant tissues during development. BBIs in group II were predominantly expressed in root tissues, while BBIs in group III were expressed most highly during the early stages of leaf, stem and spike development. Finally, genes in group IV showed low levels of expression in most tissues and included ten genes with no detectable transcripts in any assayed tissue (Fig. 8a).

We also identified a subset of wheat BBIs that exhibit stress-responsive changes in expression (Fig. 8b). The majority of the highly expressed BBIs in group I are induced in response to stripe rust and *Septoria tritici* blotch infection and are suppressed by heat stress (Fig. 8b). Several BBIs in other groups were induced by multiple biotic stresses, including some genes in group IV that were only expressed in response to stress, indicating they may play a role in general immunity. Many of the BBI genes with no detectable expression in any of the reported conditions encode proteins lacking a SP or with truncations and amino acid changes in critical domains, suggesting they may be non-functional (Fig. 8).

## Discussion

### Diverse wheat genomic resources facilitate gene family characterization studies

In this study, we identified and characterized the BBI gene family in the common wheat landrace ‘Chinese Spring’, four modern cultivars, and their extant progenitors, using HMM-based homology searches (Fig. 1). This approach incorporates position-specific alignment scores and ensemble algorithms to evaluate all possible alignments. By weighting the relative likelihood of each alignment to identify orthologous proteins against a Pfam protein database, HMM may provide greater sensitivity than other sequence-based searches to identify all members of a gene family [57]. In addition, Pfam annotations are more specific than superfamily protein groupings that are assembled in other databases, allowing for the more stringent classification of proteins. For each species, a single HMMsearch using a profile downloaded from the Pfam database was insufficient to identify all BBI proteins, likely because this general profile does not reflect species-specific diversity in this protein family [58]. A second search using a custom HMM profile built from an alignment of BBIs from the first screen yielded additional BBIs in every species analyzed, and for wheat, included 13 BBI proteins not associated with a BBI Pfam domain in their IWGSC RefSeq v1.0 gene model annotations [55]. We confirmed that each protein contained at least one BBI Pfam domain using HMMscan, although it is important to note that these sequences represent in silico predictions and the inhibitory function of each protein should be validated using biochemical assays, especially for those lacking conserved Cys residues.

Access to a greater diversity of high-quality genome assemblies for wheat will allow for more detailed gene characterization studies in this species. For example, the recent assembly of a more contiguous ‘Chinese Spring’ genome using both short and long read sequencing resolved 5799 gene duplications that were not annotated in IWGSC RefSeq v1.1 [59]. These include two BBI genes (*T4033720*, a paralog of *TraesCS3A02G046300* that is located 6 Mb downstream on the same chromosome, and *T4042195*, a paralog of *TraesCS5B02G498100* located on chromosome 3B) that were not present in the IWGSC RefSeq v1.1 assembly (Additional file 2, Table S8). Beyond ‘Chinese Spring’, an international wheat pan-genome project aims to sequence and assemble multiple common wheat genomes [60]. Among the five varieties we analyzed in this study, no two had the same complement of BBI genes (Fig. 5), although it is important to note that presence/absence variation between varieties may be the result of incomplete genome assembly. A set of fully-annotated, high-quality genome assemblies of diverse wheat varieties will be a valuable resource to characterize the full extent of natural genetic variation in wheat.

### The wheat BBI gene family underwent extensive duplication resulting in copy number variation and multi-domain proteins

Consistent with previous studies, our phylogenetic analysis shows that the BBI family is subject to widespread gene duplication events that likely occurred independently in each monocot species since they last shared a common ancestor [25, 38]. In rice, ten BBI genes are located in a 430 kb region of chromosome 1 [25], in maize, four BBIs are 200 kb apart on chromosome 3 and in barley, eleven BBIs are located within a 450 kb region of chromosome 3H (Fig. 3c, Additional file 2, Table S5). Each of these regions is syntenic with the distal region of wheat homoeologous group 3 chromosomes [61, 62], suggesting that a common mechanism associated with this region of the genome, likely conserved in all crop species, triggers gene duplication at these loci. We found some evidence of other gene duplication events within 200 kb of BBI gene clusters in wheat, including 11 genes encoding proteins annotated as “Disease resistance RPM1” on chromosome 1B and 12 genes encoding E3-Ubiquitin ligase proteins on chromosome 3D (Additional file 2, Table S9 and Table S10). However, these duplications were not shared between homoeologous chromosomes, suggesting they arose from recent duplication events, and are unlikely to be associated with BBI protein function in wheat.

One possible factor contributing to the high rate of duplications in the BBI family may be the location of gene clusters in distal telomeric regions of each chromosome (Fig. 2), which are hotspots for evolution, recombination events [63] and, in polyploid species, homoeologous exchange [64]. Characterization of the MADS-box transcription factor family in wheat revealed a positive correlation between the number of genes in a subfamily and their proximity to the telomere [65]. In barley, large segmental duplications occurred more frequently in the telomeres, and were associated with increased gene copy number variation, potentially because of higher rates of non-allelic homologous recombination in these regions [66]. However, their position alone cannot account for the extent of BBI duplication, because the genes on homoeologous group 4 and 5 chromosomes are similarly located in the telomere but did not undergo duplication in any barley or wheat genome analyzed in our study (Fig. 3c).

Although we found evidence of BBI gene duplication in all analyzed monocot genomes, this family was larger in wheat and barley due to more extensive tandem duplication events on wheat homoeologous group 1 and 3 chromosomes and barley chromosome 3H (Fig. 3). Although many of these gene duplication events had already occurred in wheat’s diploid and tetraploid progenitors, we also identified several duplication events that occurred since common wheat’s domestication (Fig. 4), demonstrating that the process driving BBI family expansion in wheat remains active. In polyploid wheat species, relaxed selection pressure arising from gene redundancy may partially account for the greater expansion of the BBI gene family [67]. However, the similar size of the BBI gene family in diploid barley and wheat progenitors shows that gene duplication occurs to a similar degree in different Triticeae species, demonstrating that polyploidy is not necessary for BBI duplication. Further studies will be required to determine the mechanism or factors driving BBI gene family expansion in the Triticeae.

Our study also revealed that BBI domain duplication, possibly originating from incomplete gene duplication followed by gene fusion or internal duplication, resulted in further diversification of encoded wheat BBI proteins, potentially enlarging the spectrum of their protease substrates (Fig. 6). Despite the high level of conservation of the P1-P1’ motif in wheat (Fig. 6), this motif is more variable in other monocots such as rice and banana [25, 38], so its importance for substrate recognition will require further analysis. Domain duplication is a common feature of BBI evolution in different plant species, including an ancient event that gave rise to the “double-headed” BBI structure conserved in dicots [25, 36, 38]. Our in silico analysis predicted that all wheat BBI proteins lack a functional second reactive motif to inhibit chymotrypsin activity (Fig. 6), consistent with analyses of other monocot BBIs [36, 37]. However, previous studies have detected chymotrypsin inhibition in protein extracts from the wheat endosperm, so it is likely that this activity is performed by a distinct family of protease inhibitors, potentially members of the cereal trypsin/α-amylase inhibitor family [68, 69].

Multi-domain monocot BBIs were previously isolated and characterized in other monocot species [25, 36, 70, 71]. The separation of all single-domain BBIs and all multi-domain BBIs in our phylogenetic tree suggests that these multi-domain BBIs were already present in the common ancestor of these grasses (Fig. 3c). In wheat, all multi-domain BBIs are located on homoeologous group 3 chromosomes (Fig. 6c-d). Our finding that BBIs on both group 1 and group 3 chromosomes underwent complete gene duplication but only the BBIs on group 3 chromosomes underwent domain duplication (Fig. 3c and Fig. 6), suggests that the mechanism of gene duplication differs between group 1 and group 3 chromosomes. Alternatively, the reduced selective pressure on BBI genes on homoeologous group 3 chromosomes (Additional file 1, Fig. S1) may result in a higher magnitude of gene expansion and an increased frequency of internal duplications giving rise to multi-domain proteins. These include three- and four-domain BBI proteins distinct in structure from any previously proposed BBI protein model (Fig. 6d). In order to determine the impact of this variation, it will be critical to identify the endogenous and exogenous interacting substrates of the BBI family.

### Functional characterization of wheat BBI genes

Gene duplication events can impact molecular evolution in different ways [72]. These include: (i) loss of protein function resulting from excessive mutation accumulation (ii) gain of protein function as a result of gene overexpression, (iii) neo- or sub-functionalization, and (iv) modulation of protein activity by duplicating and diversifying reactive sites. Our analyses indicate that the wheat BBI family potentially contains members exhibiting each of these features. Several wheat BBI genes exhibited truncations, mutations in active sites and undetectable transcript levels in all assayed tissues (Fig. 8a), suggesting they may be non-functional pseudogenes. Conversely, we also identified homoeologous BBI genes that exhibit divergent expression profiles, suggesting they may have taken on new functional roles during wheat development (Fig. 8a). Several wheat BBIs exhibit high transcript levels in the grain, suggesting they may regulate endogenous protease activity during grain development (Fig. 8a). We also identified a subset of BBIs that are transcriptionally induced in response to fungal and bacterial pathogens, consistent with previous studies in other plants [25, 51, 73], which may indicate these genes contribute to plant defense responses (Fig. 8b). One of these genes, *TraesCS1A02G021400*, is induced in response to four pathogens (Fig. 8b), and was previously identified as a candidate gene for wheat seedling resistance to tan spot [28]. It would be interesting to characterize this gene to determine its potential role in disease resistance in wheat. Another wheat BBI gene, *TraesCS1B02G025900*, was identified as a candidate defense hub gene for Type II Fusarium head blight resistance [29]. However, this BBI gene is expressed primarily in root and stem tissues and is not induced in response to any biotic stress assayed in our study (Fig. 8), suggesting it is unlikely to play a role in disease resistance. Some pathogens secrete proteases as part of their infection cycle, and in response, plants have co-evolved different classes of PIs to inhibit their activity [25]. In wheat, a greater number of BBI proteins with more numerous and diverse reactive sites may allow the wheat plant to inhibit a wider range of pathogenic protease substrate variants as part of an effective response against fungal and bacterial pathogens [25]. Identification of the protease inhibitors interacting with wheat BBIs will allow for a more detailed understanding of their mode of action. Future studies might include an analysis of the co-expression of BBIs and their protease targets during development and in response to biotic or abiotic stress. It is interesting to note that the distal area of chromosome arm 3BS, which includes a cluster of 14 BBI genes, overlaps with the Wheat Streak Mosaic Virus resistance locus *Wsm2* [74, 75]. Although there is no evidence that BBI proteins act as an R gene for virus resistance, they might function as antagonistic interacting proteins with other R proteins to trigger defense responses [33].

## Conclusions

We found that the BBI gene family in common wheat is larger than in other monocots due to a series of tandem duplication events in the telomeric regions of homoeologous group 1 and group 3 chromosomes. The increased frequency of gene duplications on homoeologous group 3 chromosomes likely gave rise to multi-domain BBI proteins with novel reactive sites. It will be important to determine the endogenous and exogenous protease substrates of individual BBIs and to identify how divergent and duplicated active sites impact their specificity and activity. Our description of this gene family in wheat will facilitate the functional characterization of individual BBI genes. Reverse genetics tools will facilitate hypothesis testing to determine the role of BBI genes in wheat development and defense responses [76], and to help identify natural genetic variation that may be valuable for elite cultivar development.

## Methods

### Identification of Bowman-Birk inhibitors in plant genomes

High and low confidence wheat protein annotations from IWGSC RefSeq v1.1 [55] were downloaded from the IWGSC sequence repository hosted by URGI (https://urgi.versailles.inra.fr/download/iwgsc/IWGSC_RefSeq_Annotations/v1.1/) and concatenated into a single FASTA file consisting of 298,774 protein sequences. Protein sequences were obtained from the reference assemblies of *Hordeum vulgare* (IBSC_v2, 236,301 protein sequences), *Brachypodium distachyon* (v3.0, 52,972 protein sequences), *Aegilops tauschii* (Aet_v4.0, 258,680 protein sequences) [77], and *Triticum urartu* (ASM34745v1, 33,483 protein sequences) [78] from Ensembl Plants (https://plants.ensembl.org/info/data/ftp/index.html). *Oryza sativa* proteins were downloaded from the Rice Genome Annotation Project (Oryza_japonica.MSUv7, 55,986 protein sequences) (RGAP, http://rice.plantbiology.msu.edu) and converted to IRGSP-1.0 gene IDs and *Zea mays* proteins (Zea_mays.B73_RefGen_v4, 131,585 protein sequences) were downloaded from MaizeGDB (https://www.maizegdb.org). *Triticum turgidum ssp. dicoccoides* wild emmer wheat ‘Zavitan’ WEWseq v2 proteins (205,916 sequences) were downloaded from https://search.datacite.org/works/10.5447/ipk/2019/0 [79].

The identification of BBI proteins in each species was performed with HMMER analysis [57] against the local protein annotation database using a three-step approach outlined in Fig. 1. First, we performed an HMMsearch using the HMM profile for the Bowman-Birk protease inhibitor family (Pfam: PF00228) which was downloaded from Pfam 32.0 [58] using an E-value threshold of 1e^− 5^. We next aligned the BBI protein sequences identified from the first step using HMMalign and built a new HMM profile based on the multiple alignment using HMMbuild. We used the new generated HMM profile to conduct a second HMMsearch against the same species-specific protein databases. Finally, we examined the list of BBI proteins for the presence of a BBI Pfam domain (PF00228) using HMMscan with an E-value threshold of 0.05. Proteins that contained the Pfam domain were classified as BBI. We then performed alignment of the identified BBI protein sequences from all species with MAFFT [80] and noticed that several BBIs were predicted to lack a signal peptide due to misannotation of the methionine start codon. We manually curated the position of the N-terminal start codon of several BBIs from *T. aestivum*, *Ae. tauchii*, *T. urartu*, *T. dicoccoides* and *H. vulgare* to match homologous sequences. Full curation details are provided in Additional file 2, Table S2, and includes details of BBI proteins with N-terminal truncations likely caused by point mutations. The curated sequences were used in all subsequent analyses.

### Chromosomal locations and homology identification

All identified wheat BBIs were mapped to the IWGSC Refseq v1.1 genome assembly to identify their chromosomal location [55]. To determine homologous relationships between genes, we performed all-to-all BLAST using the 57 proteins as queries and applied an E-value threshold of 1e^− 10^. Putative paralogs or homoeologs were defined as homologous BBIs with a BLASTP e-value <1e^− 10^ and identity > 75% on the same or homoeologous group chromosome, respectively. This approach was also used to identify orthologous relationships between BBIs in common wheat and progenitor genomes. The synteny and homologous relationship of wheat BBI genes were visualized with Circos plot using R shinyCircos [81]. Each chromosome was divided into telomere (R1/R3), centromere (C) and R2 segments according to information from the IWGSC RefSeq v1.1 genome assembly [55]. The distance of wheat BBIs and other high- and low-confidence gene models were mapped to individual chromosomes using the R Sushi package plotBed function [82].

We calculated Ka/Ks ratios using an online tool hosted by the computational biology unit (CBU http://services.cbu.uib.no/tools/kaks) using the coding sequence of each common wheat BBI gene. We excluded one BBI (TraesCS3D02G035800) from the Ka/Ks ratio analysis due to a premature termination codon in its coding sequence. The remaining 56 BBIs were grouped according to their chromosome and used to construct phylogenetic trees and calculate the pairwise Ka/Ks ratio for each branch.

### Alignment and phylogenetic analysis

We performed multiple sequence alignments using Clustal Omega using full-length BBI protein sequences identified in all species. Model selection was conducted with IQ-TREE using the lowest Bayesian information criterion (BIC) as WAG+G4 model [83]. We constructed the phylogenetic tree using the selected model with 1000 ultrafast bootstrap replicates UFBoot2 [84, 85]. The resulting tree was visualized and annotated with the R package ggtree v2.0.4 [86]. The domain model type for BBIs in grasses were determined manually by comparing the number and position of Cys residues to the model proposed by Mello et al. [36].

### Identification of BBI on homoeologous group 3 chromosomes in different wheat varieties

The draft genome assembly for four common wheat varieties ‘Jagger’ (U.S.A, winter growth habit), ‘Julius’ (Germany, winter), ‘Landmark’ (Canada, spring), and ‘Mace’ (Australia, spring) were downloaded from the 10+ Wheat Genomes Project https://webblast.ipk-gatersleben.de/downloads/wheat/ and used to build local BLAST databases. We then used the full-length protein-coding sequences of each BBI from ‘Chinese Spring’ as queries and performed BLAST against the genomes of each wheat variety to identify their chromosomal position [87]. The position of each BBI gene in these varieties was cross-referenced with GFF files to identify the corresponding gene ID provided by the 10+ Wheat Genome Project [60]. To identify BBIs present in these varieties but absent from the ‘Chinese Spring’ assembly, the corresponding genomic region spanning all BBI genes on homoeologous group 1 and group 3 chromosomes from each wheat variety were extracted locally using the bedtools getfasta command for *ab initio* gene prediction [88]. The open reading frame (ORF) and putative gene model for each extracted DNA fragment was predicted with OrfM-0.7.1 [89]. To determine whether predicted gene models contain a functional BBI domain, all predicted ORFs were scanned with HMMscan using an e-value cutoff of 0.05 and those BBIs with PF00228 domains were retained. After exclusion of common orthologs in other varieties, the unique BBIs in each variety were named using the first two letters of the cultivar name, followed by chromosome number, and ordered by their relative position on that chromosome. For example, JA_3A-1 represents the first unique BBI on ‘Jagger’ chromosome 3A. The BBI protein sequences from all varieties were then used to construct a phylogenetic tree with IQ-TREE using the WAG+G4 model with 1000 ultrafast bootstrap replicates UFBoot2 [84, 85]. The resulting tree was visualized and annotated with the R package ggtree v2.0.4 [86].

### Gene structure analysis of the functional domains and motifs

The complete genomic, CDS and amino acid sequences, as well as gene feature information of all BBIs identified were downloaded from IWGSC RefSeq v1.1 [55]. Schematic representation of the exon-intron organization of wheat BBIs was conducted by comparing the CDS and the corresponding genomic sequences using Gene Structure Display Server 2.0 [90]. To find conserved Cys-rich domains, the amino acid sequence for the functional domains of all identified BBIs in wheat, by aligning amino acid sequences between the first and last conserved Cys residue in each domain using MAFFT v7 for multiple sequence alignment [80]. All sequences were analyzed using Signal P v5.0 [91] to predict the presence of N-terminal SP and for potential cleavage sites.

### Gene expression analysis

The expression data for wheat BBI genes in five tissues (spike, root, leaf, grain and stem) at three different developmental stages from hexaploid wheat landrace ‘Chinese Spring’ [92] and under abiotic stress (heat and drought) condition at the one-week-old seedling stage [93] were mapped to the IWGSC RefSeq v1.1 genome and processed into TPM values as described previously [94]. Separately, we downloaded several biotic stress expression datasets as TPM from the online wheat expression browser expVIP [56], including studies on fusarium head blight [95, 96], stripe rust [97, 98], powdery mildew [98], fusarium crown rot [99], *Septoria tritici* blotch [100, 101] and PAMP elicitors [102]. For each pathogen, we calculated the log2 fold change of the transcript abundance for each treated sample compared to mock controls or samples at time zero at each time point and averaged the values of all time points. Heatmaps for tissue specific time course expression were constructed using log2 transformed TPM values with the R package pheatmap v1.0.12. Genes were clustered according to their expression level (metric, Euclidian; method, complete) and grouped by their chromosome type.

## Supplementary Information

**Additional file 1: Fig. S1.** Ka/Ks phylogenetic tree of common wheat BBIs separated by chromosome. The values on each branch indicate the ratio for that pair of genes. Branches and values greater than one are highlighted in red. **Fig. S2.** Phylogenetic tree of BBIs identified in common wheat landrace ‘Chinese Spring’ and four common wheat varieties (‘Jagger’, ‘Mace’, ‘Landmark’ and ‘Julius’) on **a** homoeologous group 1, 4, and 5 chromosomes and **b** homoeologous group 3 chromosomes**.** The trees were built with the model (WAG+G4) which has the lowest BIC value using 1000 bootstrap replications. Only bootstrap support values below 95 are indicated on the tree. Genes are color-coded based on wheat variety. **Fig. S3.** Structural characterization of common wheat BBIs. **a** Phylogenetic tree of 57 wheat BBI genomic sequences. The alignment was conducted with IQ-TREE to predict best fit model for nucleic acid with the lowest BIC value. Gene names are color coded to indicate different clades which were grouped based on their nucleic acid structure. **b** Intron-exon structure of each BBI gene predicted by comparison of CDS and gDNA sequence. Blue rectangles indicate untranslated regions, black lines indicate introns and yellow rectangles indicate exons. **c** Functional domain discovery, the Bowman-Birk domain prediction was conducted by NCBI-CDD to look for smart00269 (Bowman-Birk type protease inhibitor from SMART database). Blue rectangles indicate BBI functional domains and black lines indicate other amino acids.)

**Additional file 2: Table S1.** List of 62 common wheat BBIs in the IWGSC RefSeq v1.1 genome assembly, including five additional genes that were excluded due to the lack of a complete BBI domain. Information includes their gene position (Gene ID based on IWGSC Refseq v1.1 gene models, name based on their homoeologous relationships, chromosome locations and order), gene structure and features (number of exons and BBI domains, BBI domain evolutionary model types [36], amino acids at P1-P1` motif position, number of complete BBI domains with all required Cys residues, protein length and molecular weight), signal peptide prediction (SP prediction as signal peptide or other, prediction confidence, predicted cleavage site position, and + = present, − = absent), pseudogene prediction (T = True, F = False), and log_2_TPM values of expression during development and log_2_ fold-change TPM of biotic and abiotic stress expression datasets. **Table S2.** List of BBI genes in *T. aestivum, Ae. tauschii, T. urartu, T. dicoccoides*, and *H. vulgare* for which manual curation was performed. Details of the position and confidence level of the signal peptide site are included for both the original predicted sequence and the manually curated sequence. Full details of the manual curation are provided in column K, which have been corrected for the initiation codon. All curated sequences have a signal peptide prediction greater than 0.97. BBI genes with abnormal N-terminal truncation were also listed in column G. **Table S3.** List of six putative wheat BBIs identified in previous studies. Information includes their original and alternative gene names, corresponding protein ID in the UniProt database, e-value for HMMscan of the BBI domain, protein sequences documented in the UniPort database, complete protein sequences based on their annotation in the IWGSC RefSeq v1.1 genome assembly and citations for the studies where these proteins were originally reported. **Table S4.** Common wheat BBI homoeologous groups divided by chromosome. **Table S5.** List of BBIs identified from *O. sativa, Z. mays, B. distachyon, H. vulgare, Ae. tauschii, T. urartu* and *T. dicoccoides.* Information includes species, gene number named by order of the gene ID from the source model, alternative name and the citation for where the name was first described, gene ID, chromosome position, BBI domain type, protein length, number of BBI domains, source of genome assembly and gene ID converter from IRGSP-1.0 to MSU for rice BBIs. For BBIs without alternative names and with uncharacterized model types, we used ‘-’ symbol. **Table S6.** Homologous relationships of BBIs in common wheat compared to *T. urartu* (AA genome), *Ae. tauschii* (DD genome) and *T. dicoccoides* (AABB genomes). Orthologous genes are presented in the same row. BBIs with uncharacterized homologous relationships were placed in separate rows and labelled “ungrouped”. **Table S7.** List of BBIs identified in common wheat cultivars ‘Jagger’, ‘Landmark’, ‘Julius’ and ‘Mace’. Information includes their gene ID according to the 10+ wheat genome project annotation [60], chromosome and positions, and their projections in ‘Chinese Spring’ where available. BBI genes present in some cultivars but absent from ‘Chinese Spring’ were named based on the cultivar (e.g. JA means ‘Jagger’, JU means ‘Julius’) followed by their chromosome and the physical order on that chromosome based on our de novo ORF prediction. For example, *JA_1D-1* refers to the first BBI gene on ‘Jagger’ chromosome 1D that is absent from the ‘Chinese Spring’ reference assembly. **Table S8.** List of two common wheat BBIs identified in the “Triticum 4.0” assembly of ‘Chinese Spring’, but absent from the IWGSC RefSeq v1.1 assembly. Information includes their gene name, chromosomal location, corresponding orthologous gene from the IWGSC RefSeq v1.1 assembly, gene structure and features (exon and domain numbers, model types, P1-P1` motif residues, protein length and molecular weight) and signal peptide prediction. **Table S9.** Functional annotation and genomic position of all genes 200 kb upstream and downstream of BBI clusters on homoeologous group 1 and 3 chromosomes. Information includes gene ID for both high and low confidence genes, their location on each chromosome, and their functional annotation and Pfam domains based on IWGSC RefSeq v1.1 gene models [55]. BBI genes identified in our study are highlighted in red and genes annotated as other trypsin inhibitors are highlighted in blue. **Table S10.** Number of genes sharing functional annotation terms from IWGSC RefSeq v1.1 gene models 200 kb upstream and downstream of BBI clusters on homoeologous group 1 and 3 chromosomes. The number of BBIs on each chromosome is highlighted in red. Gene number is based on descriptive annotations from gene models. Because some BBI genes identified in our study are annotated as ‘trypsin inhibitor’ in these gene models, there is a slight discrepancy between the number of BBI genes described in this table and the total number of BBIs.

## Data Availability

All sequence data analyzed in this project are available in public databases. Wheat DNA sequences, accessions and functional information described in Additional file 2, Tables S1 and S9 were retrieved from the IWGSC RefSeq v1.1 genome assembly from https://urgi.versailles.inra.fr/download/iwgsc/IWGSC_RefSeq_Annotations/v1.1/. Protein sequences from the assemblies of *Hordeum vulgare* (IBSC_v2), *Brachypodium distachyon* (v3.0), *Aegilops tauschii* (Aet_v4.0), *Triticum urartu* (ASM34745v1) and *Zea mays* (Zea_mays.B73_RefGen_v4) were downloaded from http://plants.ensembl.org/info/data/ftp/index.html. Protein from *Oryza sativa* (Oryza_japonica.MSUv7) were downloaded from http://rice.plantbiology.msu.edu/pub/data/Eukaryotic_Projects/o_sativa/annotation_dbs/pseudomolecules/version_7.0/all.dir/. *Triticum turgidum ssp. dicoccoides* wild emmer wheat ‘Zavitan’ WEWseq v2 proteins were downloaded from https://search.datacite.org/works/10.5447/ipk/2019/0. The protein sequences from different plant species detailed in Additional File 2, Tables S2, S5 and S6 were all derived from the above repositories. The six putative BBI protein sequences described in Additional File 2, Table S3 were downloaded from UniProt (https://www.uniprot.org/). Gene ID and chromosomal positions in Additional File 2, Table S7 were downloaded from https://webblast.ipk-gatersleben.de/downloads/wheat/gene_projection/. Triticum 4.0 genome gene accessions described in Additional File 2, Table S8 were downloaded from https://github.com/TriticumAestivum/Annotation [59].

## Abbreviations

BBI: Bowman-birk inhibitor
BLAST: Basic local alignment search tool
cDNA: Complementary deoxyribose nucleic acid
CDS: Coding sequence
Cys: Cysteine
GFF: General feature format
HMM: Hidden markov model
IWGSC: International wheat genome sequencing consortium
kb: Kilobase pair
kDa: Kilo dalton
Mb: Megabase pair
ORF: Open reading frame
PAMP: Pathogen-associated molecular patterns
Pfam: Protein family
PI: Protease inhibitor
SP: Signal peptide
TPM: Transcripts per million
