# Supplementary material for: Extensive structural variation in the Bowman-Birk inhibitor family in common wheat (Triticum aestivum L.)
Source: BMC Genomics. 2021 Mar 25;22:218. doi: 10.1186/s12864-021-07475-8 (PMC7995804; doi:10.1186/s12864-021-07475-8)

Extensive structural variation in the Bowman-Birk inhibitor family in common wheat (*Triticum aestivum* L.)

Additional file 1, Figures S1-S3 (.pdf)

Figure S1: Ka/Ks phylogenetic tree of common wheat BBIs separated by chromosome. The values on each branch indicate the ratio for that pair of genes. Branches and values greater than one are highlighted in red.

Figure S2: Phylogenetic tree of BBIs identified in common wheat landrace 'Chinese Spring' and four common wheat varieties ('Jagger', 'Mace', 'Landmark' and 'Julius') on **a** homeologous group 1, 4, and 5 chromosomes and **b** homeologous group 3 chromosomes**.** The trees were built with the model (WAG+G4) which has the lowest BIC value using 1000 bootstrap replications. Only bootstrap support values below 95 are indicated on the tree. Genes are color-coded based on wheat variety.

Figure S3: Structural characterization of common wheat BBIs. **a** Phylogenetic tree of 57 wheat BBI genomic sequences. The alignment was conducted with IQ-TREE to predict best fit model for nucleic acid with the lowest BIC value. Gene names are color coded to indicate different clades which were grouped based on their nucleic acid structure. **b** Intron-exon structure of each BBI gene predicted by comparison of CDS and gDNA sequence. Blue rectangles indicate untranslated regions, black lines indicate introns and yellow rectangles indicate exons. **c** Functional domain discovery, the Bowman-Birk domain prediction was conducted by NCBI-CDD to look for smart00269 (Bowman-Birk type protease inhibitor from SMART database). Blue rectangles indicate BBI functional domains and black lines indicate other amino acids.

Figure S1: Ka/Ks phylogenetic tree of common wheat BBIs separated by chromosome. The values on each branch indicate the ratio for that pair of genes. Branches and values greater than one are highlighted in red.

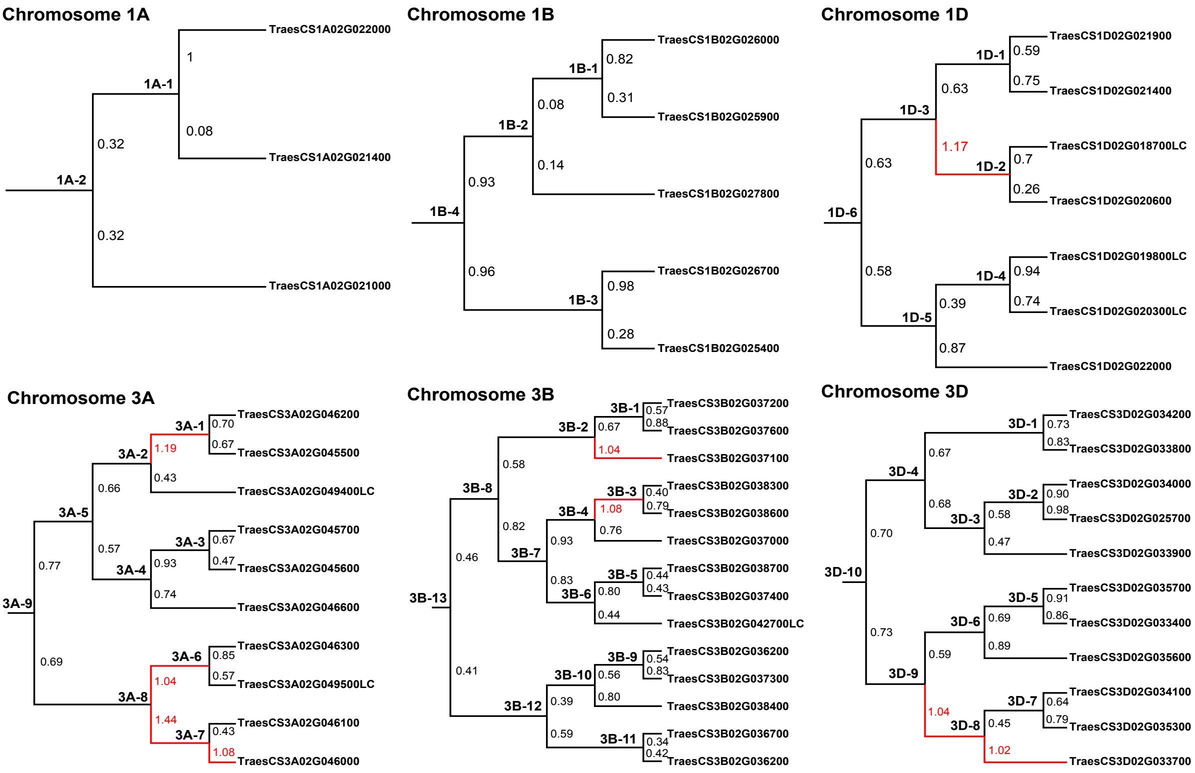

Figure S2: Phylogenetic tree of BBIs identified in common wheat landrace ‘Chinese Spring’ and four common wheat varieties (‘Jagger’, ‘Mace’, ‘Landmark’ and ‘Julius’) on **a** homeologous group 1, 4, and 5 chromosomes and **b** homeologous group 3 chromosomes. The trees were built with the model (WAG+G4) which has the lowest BIC value using 1000 bootstrap replications. Only bootstrap support values below 95 are indicated on the tree. Genes are color-coded based on wheat variety.

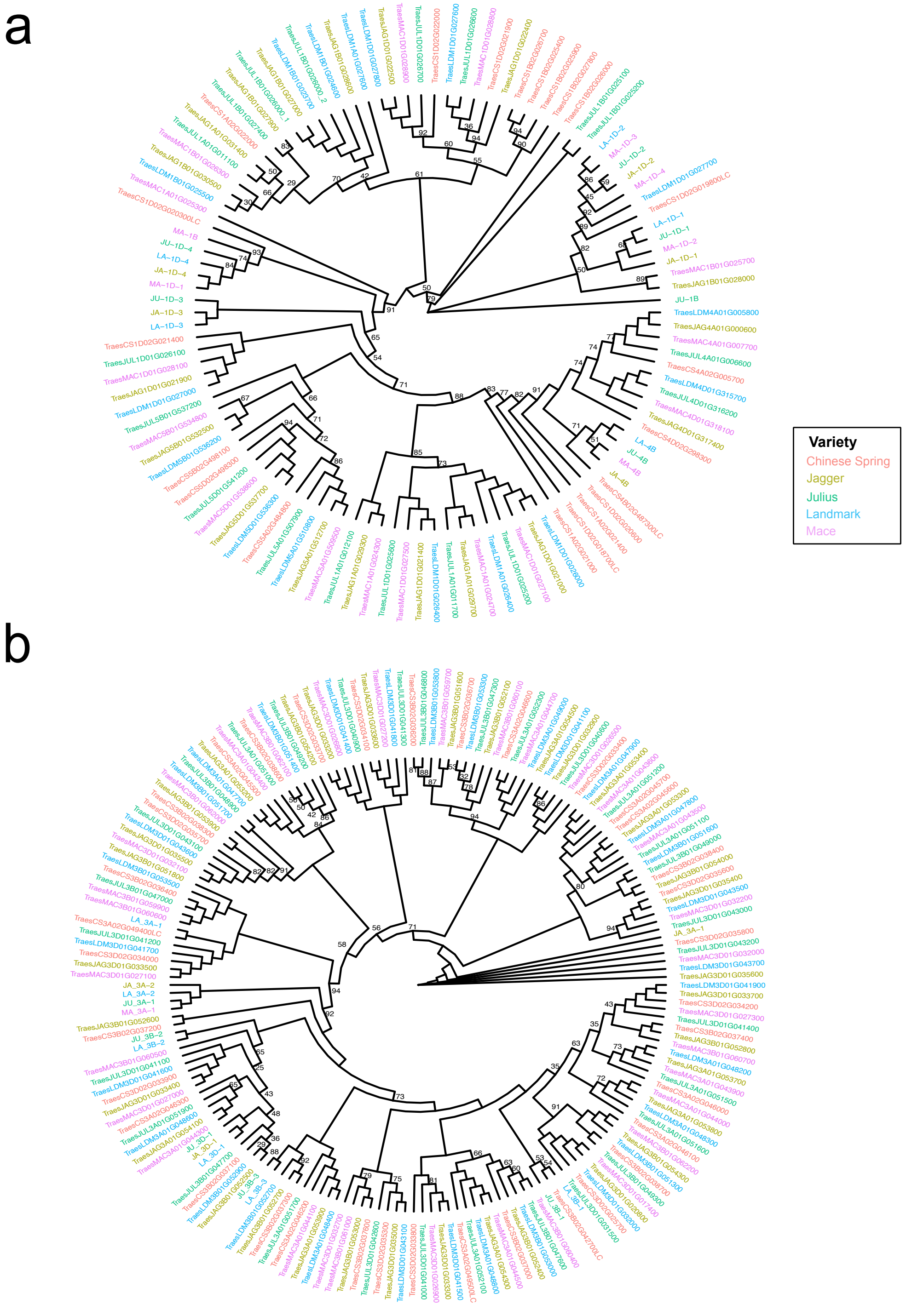

Figure S3: Structural characterization of common wheat BBIs. **a** Phylogenetic tree of 57 wheat BBI genomic sequences. The alignment was conducted with IQ-TREE to predict best fit model for nucleic acid with the lowest BIC value. Gene names are color coded to indicate different clades which were grouped based on their nucleic acid structure. **b** Intron-exon structure of each BBI gene predicted by comparison of CDS and gDNA sequence. Blue rectangles indicate untranslated regions, black lines indicate introns and yellow rectangles indicate exons. **c** Functional domain discovery, the Bowman-Birk domain prediction was conducted by NCBI-CDD to look for smart00269 (Bowman-Birk type protease inhibitor from SMART database). Blue rectangles indicate BBI functional domains and black lines indicate other amino acids.

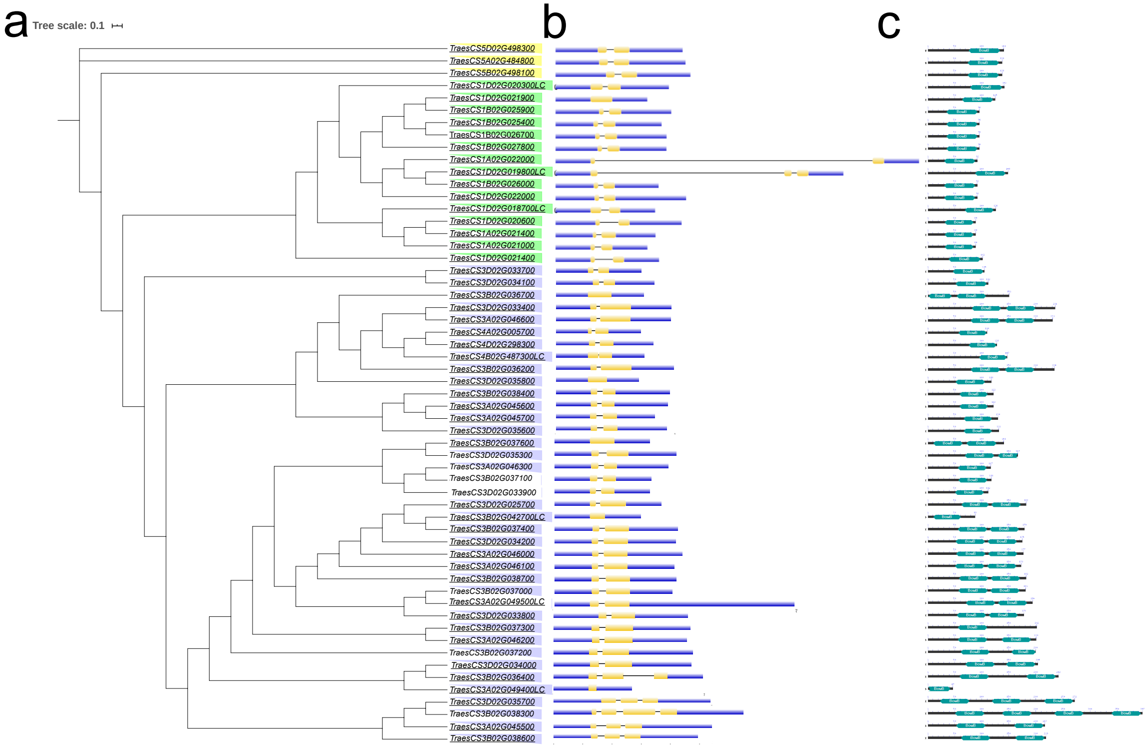

Supplement: Supplementary file 1 — Additional file 1: Fig. S1. Ka/Ks phylogenetic tree of common wheat BBIs separated by chromosome. The values on each branch indicate the ratio for that pair of genes. Branches and values greater than one are highlighted in red. Fig. S2. Phylogenetic tree of BBIs identified in common wheat landrace ‘Chinese Spring’ and four common wheat varieties (‘Jagger’, ‘Mace’, ‘Landmark’ and ‘Julius’) on a homoeologous group 1, 4, and 5 chromosomes and b homoeologous group 3 chromosomes. The trees were built with the model (WAG+G4) which has the lowest BIC value using 1000 bootstrap replications. Only bootstrap support values below 95 are indicated on the tree. Genes are color-coded based on wheat variety. Fig. S3. Structural characterization of common wheat BBIs. a Phylogenetic tree of 57 wheat BBI genomic sequences. The alignment was conducted with IQ-TREE to predict best fit model for nucleic acid with the lowest BIC value. Gene names are color coded to indicate different clades which were grouped based on their nucleic acid structure. b Intron-exon structure of each BBI gene predicted by comparison of CDS and gDNA sequence. Blue rectangles indicate untranslated regions, black lines indicate introns and yellow rectangles indicate exons. c Functional domain discovery, the Bowman-Birk domain prediction was conducted by NCBI-CDD to look for smart00269 (Bowman-Birk type protease inhibitor from SMART database). Blue rectangles indicate BBI functional domains and black lines indicate other amino acids.) [file 12864_2021_7475_MOESM1_ESM.pdf]
